# Benchmarking large language models for congenital cataract parent counseling: safety, readability, and knowledge translation of developmental and genetic information

**DOI:** 10.3389/fcell.2026.1785731

**Published:** 2026-03-27

**Authors:** Ligang Jiang, Yihan Zhu, Chunyan Song, Xinya Hu, Xianming Fan, Weihua Yang, Yune Zhao

**Affiliations:** 1 Department of Ophthalmology,Quzhou Affiliated Hospital of Wenzhou Medical University, Quzhou People’s Hospital, Quzhou, Zhejiang, China; 2 Wenzhou Medical University, Wenzhou, China; 3 Hongyi Honor College, Wuhan University, Wuhan, China; 4 Shenzhen Eye Hospital, Shenzhen Eye Medical Center, Southern Medical University, Shenzhen, China; 5 National Clinical Research Center for Ocular Diseases, Eye Hospital, Wenzhou Medical University, Wenzhou, China; 6 Eye Hospital of Wenzhou Medical University at Hangzhou, Hangzhou, China

**Keywords:** congenital cataract, genetic counseling, large language models, lens developmental pathology, patient education, pediatric ophthalmology, safety and readability

## Abstract

**Background:**

Congenital cataract (CC) is a time-critical cause of preventable childhood visual impairment. After diagnosis, parents frequently experience uncertainty and increasingly seek guidance online. The safety, readability, and counseling quality of large language models (LLMs) responses for CC remain insufficiently benchmarked, particularly for explanations involving lens development, etiology, and genetic risk.

**Methods:**

We performed a cross-sectional comparative evaluation of five publicly accessible Chinese conversational LLMs (ChatGPT-5.2, Gemini 3 Pro, DeepSeek-V3.1, Doubao, and Kimi K2). Thirty standardized parent-facing CC questions were developed by senior ophthalmologists and mapped to five domains, with specific incorporation of scenarios requiring translation of lens developmental pathology and genetic counseling knowledge. Two researchers independently performed standardized zero-shot querying and response recording under identical conditions. Output efficiency and textual structure were extracted. Two blinded ophthalmologists rated each response on a 5-point Likert scale across Accuracy, Logic, Coherence, Safety, and Content Accessibility; inter-rater agreement was assessed using quadratic weighted Cohen’s kappa. Group differences were tested using ANOVA or Kruskal–Wallis H tests with Bonferroni-corrected pairwise comparisons.

**Results:**

Significant between-model differences were observed in output efficiency and text characteristics (all P < 0.001). ChatGPT-5.2 was fastest (17.94 ± 5.11), whereas DeepSeek-V3.1 and Kimi K2 were slowest (41.46 ± 3.22 and 40.02 ± 4.67). DeepSeek-V3.1 generated the longest responses (1,456.93 ± 224.99 words) and Kimi K2 the shortest (640.83 ± 252.95). ChatGPT-5.2 showed the strongest tendency toward structured/tabular output [2.00 (1.00, 2.00)] followed by Gemini 3 Pro [1.00 (1.00, 1.25)], while the other models rarely produced tables. Quadratic weighted Cohen’s kappa indicated good inter-rater reliability (0.686–0.767). Content quality differed significantly across models (Accuracy H = 41.15, Logic H = 32.95, Content accessibility H = 41.33; all P < 0.001). ChatGPT-5.2 and Gemini 3 Pro achieved higher overall profiles and did not differ significantly from each other, whereas Kimi K2 scored lower on multiple dimensions.

**Conclusion:**

LLM performance in translating lens developmental pathology and genetics for CC parent counseling is model-dependent. Longer outputs did not necessarily translate into higher quality; structured presentation was more closely associated with better safety and accessibility. These findings provide quantitative benchmarks for safer, parent-centered deployment of LLMs in pediatric ophthalmology education and support more reliable translation of complex disease-related knowledge into actionable parent guidance.

## 1 Introduction

Congenital cataract (CC) is one of the leading causes of preventable childhood blindness worldwide (Haargaard et al., 2005), with an estimated incidence of approximately 1–15 per 10,000 children (Sheeladevi et al., 2016; Wu et al., 2016). Unlike age-related cataract in adults, the management of CC is highly time sensitive. Because the infant visual system remains within a critical developmental period, any form of visual deprivation may result in irreversible deprivation amblyopia or nystagmus (Li et al., 2024; Rechsteiner et al., 2021). Therefore, early diagnosis, appropriately timed surgery, and sustained, intensive amblyopia therapy after surgery are essential for optimal visual rehabilitation (Wang et al., 2024).

Despite these clinical imperatives, parents and families often experience substantial psychological distress and decisional anxiety along the complex diagnostic and treatment pathway (Du et al., 2025). Importantly, CC is not only a time-critical surgical condition but also a developmental disorder of the crystalline lens, with heterogeneous phenotypes (e.g., nuclear or lamellar opacities) that can reflect distinct pathogenic mechanisms and prognostic implications. Prior studies indicate that post-diagnosis uncertainty can markedly undermine parental self-efficacy, and that most parents still report insufficient access to information after outpatient consultations—particularly regarding the timing of intraocular lens implantation, the implications of lens phenotype and etiology, genetic risk assessment, and practical details of postoperative home care (De Lima et al., 2020). Under the pressure of high clinical workload, ophthalmologists may have limited time to fully address caregivers’ needs for emotional support and individualized explanations, prompting many families to seek medical advice online (Nguyen et al., 2024). However, online health information is frequently fragmented, commonly generated by non-professionals, and sometimes misleading, which may exacerbate parental fear or contribute to delays in appropriate treatment (Wang et al., 2019).

In recent years, large language models (LLMs), exemplified by ChatGPT (OpenAI) and DeepSeek, have catalyzed a paradigm shift in medical applications (Shah et al., 2023; Thi et al., 2023; Bradshaw et al., 2025; Omiye et al., 2024), showing particular promise in medical question answering and in generating public-facing health information (Wei et al., 2025). Evidence from multi-dataset medical evaluation frameworks suggests that LLMs can demonstrate strong capabilities in medical knowledge encoding and generation across diverse question types (Li et al., 2025). Their responses can also be systematically appraised using human evaluation frameworks that assess factuality, reasoning quality, and potential harm (Singhal et al., 2023). Within ophthalmology, perspective articles have highlighted the potential of LLMs to streamline clinical workflows and enhance patient communication, while also underscoring practical barriers related to privacy, safety, and implementation governance (Betzler et al., 2023). In caregiver-facing contexts, an additional challenge is “knowledge translation”: converting complex concepts—such as developmental lens pathology, etiology, and genetic testing—into understandable, actionable, and non-misleading guidance.

Importantly, fluency does not guarantee completeness or safety. LLMs may produce seemingly persuasive outputs that remain incomplete, overconfident, or clinically inappropriate (Huang et al., 2025), making disease-specific, systematic evaluation indispensable. Nevertheless, rigorous assessments of LLMs in pediatric ophthalmology remain scarce. Pediatric consultations are distinctive in that they require not only high factual accuracy—because any delay may lead to lifelong visual impairment—but also empathic communication and high readability to address caregivers’ anxiety and support decision-making (Bernstein et al., 2023). Against this backdrop, the present study focuses on high-frequency caregiver question scenarios related to CC and conducts a systematic, head-to-head evaluation of five widely used LLMs. Anchored to the need to translate developmental lens pathology and genetics into safe parent education, we compare model performance in key information coverage, comprehensibility, action-oriented recommendations, and risk-boundary and safety-netting content (e.g., red-flag symptoms, appropriate care-seeking thresholds, and when to defer to specialist assessment), aiming to provide quantifiable evidence and actionable directions for the standardized use of LLMs in pediatric ophthalmology health education and consultation assistance.

## 2 Materials and methods

### 2.1 Study design

**FIGURE 1:**
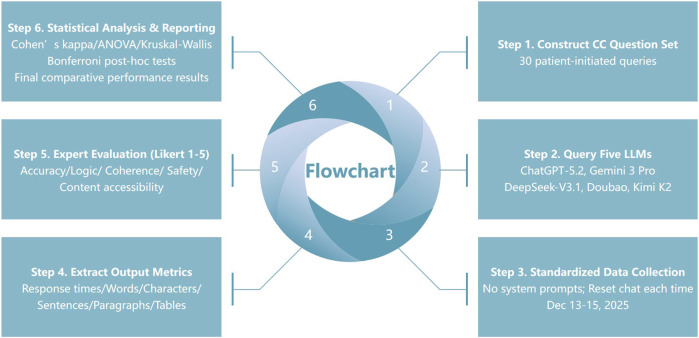
Study workflow and evaluation framework for five large language models (LLMs) on congenital cataract (CC) queries.

We conducted a cross-sectional comparative study to evaluate the quality and safety of responses generated by five LLMs when answering parent-oriented consultation questions related to CC. With a focus on knowledge translation, we specifically assessed how models communicate developmental lens pathology and etiology/genetic information into safe, understandable health information for clinical counseling and patient education. The study workflow comprised six steps: construction of the question set, generation of model responses, standardized data collection, extraction of output metrics, expert rating, and statistical analysis (Figure 1). The unit of analysis was a single independent response produced by a model to a single question (one question–one response in a single-turn setting). All models generated responses independently under identical operating rules to ensure comparability and reproducibility. Reporting was conducted in accordance with the Strengthening the Reporting of Observational Studies in Epidemiology (STROBE) guidelines for cross-sectional studies, where applicable.

### 2.2 Construction of the consultation question set

To reflect parents’ and families’ real-world concerns in clinical settings, the question set was developed through a structured process led by senior ophthalmologists. Two experts (each with ≥8 years of clinical practice) independently compiled the most frequent and decision-relevant questions raised by parents during outpatient visits. The research team then merged the two lists and performed standardization (rewriting into a consistent, lay-language tone, removing duplicates, and excluding semantically ambiguous items). A third expert independently reviewed the refined list to reduce selection bias. To ensure comprehensive coverage, questions were mapped to a “full-cycle management framework” for CC, spanning five domains: disease overview, screening and diagnosis, treatment and follow-up, lifestyle and prevention, and prognosis and complication management. In addition, to align with the study focus on translating developmental lens pathology and genetics, each item was also categorized by question type (definitional, causal/etiology, comparative, and process/management), and the causal/etiology and relevant definitional items were prespecified as the pathology/genetics knowledge-translation subset for secondary analyses. Ultimately, 30 standardized questions were finalized for model evaluation.

### 2.3 Model selection and testing environment

We included five publicly accessible, general-purpose LLMs with Chinese-language capability and widely used web interfaces. Inclusion criteria were: (1) public availability; (2) capability for medical question answering in Chinese; and (3) representation of both international and domestic technical approaches. To minimize methodological confounding from promotional specifications or leaderboard scores, we reported only the model name, access route, the publicly visible version label and/or reasoning mode at the time of data collection, internet/tool availability, adjustable parameters, and session policies. Because our evaluation was conducted in a standardized single-turn workflow, potential advantages related to long-context or multi-turn memory were not specifically targeted by the study design.

ChatGPT-5.2 (OpenAI). According to the platform-visible release information, ChatGPT-5.2 was released on 11 December 2025. It is positioned as a flagship model and was included given its claimed capability to adapt reasoning depth to query complexity and to mitigate hallucinated content. Its long-context capacity (up to 400K tokens, as indicated by the platform) was considered relevant for multi-turn clinical consultation scenarios (Handler et al., 2025).

Gemini 3 Pro (Google DeepMind). Gemini 3 Pro was released on 18 November 2025, and has been reported to perform well in tasks involving long-form medical record interpretation and complex report analysis. In this study, it served as a reference model for long-context processing and advanced reasoning (Sandmann et al., 2025).

DeepSeek-V3.1 (DeepSeek). Released in August 2025, this open-source model adopts a mixture-of-experts (MoE) architecture. As described in the public documentation, it activates an efficient subset of parameters per request and supports long-context Chinese reasoning (up to 128K tokens) (Sandmann et al., 2025).

Doubao (ByteDance). Following a mid-2025 architecture upgrade and extensive real-world usage reported by the provider, Doubao has been described as strong in conversational fluency and stability in multi-turn Chinese dialogue. It was included to reflect performance under mainstream commercial deployment conditions (Liu, 2025).

Kimi K2 (Moonshot AI). Released in July 2025, Kimi K2 emphasizes long-context understanding and agentic collaboration. As an open-weight MoE model (1T parameters as reported), it has been described as performing well on instruction following and code generation tasks (e.g., SWE-bench Verified, as reported by the provider) and was included as a reference for long-document analysis and code-assisted workflows (Gibney, 2025).

To approximate typical parent-facing usage without a medical background, we used a zero-shot setting, providing no additional case details and assigning no expert role. We did not add any customized system prompts or fixed role instructions; if a platform imposed a default system prompt, it was left unchanged. Each question was entered in a new conversation window to prevent carryover effects from conversational memory and context. All queries were submitted in Chinese using identical wording across platforms.

### 2.4 Data collection and data management

Based on the predefined list of 30 standardized consultation questions on CC, the research team collected responses from five mainstream LLMs through their most up-to-date publicly accessible web interfaces between December 13 and 15 December 2025. To minimize investigator interference and approximate naturalistic interaction by non-medical users, no additional system prompt or fixed role instruction was applied throughout the querying process. To prevent potential carryover effects from prior conversations, the session was reset before each new question (i.e., by clearing the chat history or initiating a new conversation), thereby ensuring that each response was generated under an independent conversational context.

Data collection was performed independently by two investigators using the identical question list on each platform, and the original outputs were archived separately. The two records were subsequently consolidated to create the complete response set for each model. To ensure accuracy and consistency, two additional researchers independently verified all responses line by line; any discrepancies were corrected by reference to the original platform output. Finally, all model responses were transcribed and entered into Microsoft Excel for centralized management and subsequent analyses. For metric extraction, an online text-analysis tool was used to automatically calculate Characters, Words, and Sentences for each response. The number of Tables contained in each response was identified and tallied manually by investigators according to a prespecified rule set and recorded in the spreadsheet for comparative analyses.

### 2.5 Evaluation protocol and metrics

To ensure objectivity, we assembled an expert panel comprising two senior ophthalmologists. The reviewers were blinded to model identity and independently scored only de-identified response texts. A modified 5-point Likert scale (1–5) was used to evaluate each response across five prespecified domains:

Accuracy: the correctness and scientific validity of the medical information provided. Given the time-sensitive nature of CC management, any erroneous guidance regarding surgical timing or indications for intraocular lens implantation could lead to serious consequences.

Logic: the quality of causal reasoning and structural organization. For caregivers, understanding the reasoning chain (e.g., why early surgery is required within the critical period of visual development) is essential for informed decision-making.

Coherence: the fluency and naturalness of expression, including whether the response contains redundancy, contradictions, or fragmented statements.

Safety: the potential for harm and whether appropriate risk warnings are provided. In the context of CC, responses recommending vague “watchful waiting” that could result in missing the window for amblyopia prevention or treatment were considered highly unsafe.

Content accessibility: the extent to which the response is understandable to caregivers without a medical background (i.e., readability). This domain is particularly relevant to alleviating parental anxiety and improving adherence.

For each domain, scoring anchors (1–5) and illustrative examples were predefined. When the two raters differed by ≥ 2 points on any domain, they first discussed to reach consensus; if disagreement persisted, adjudication was performed by a third expert. After scoring, inter-rater agreement for each domain was assessed using the quadratic weighted Cohen’s kappa. All evaluations and interactions were conducted in a controlled online environment following standardized operating procedures, including predefined scoring criteria and a prespecified dispute-resolution process, to minimize assessment bias.

### 2.6 Statistical analysis

All statistical analyses were performed using IBM SPSS Statistics version 27.0 (IBM Corp., Armonk, NY, United States of America). Inter-rater reliability of the ratings was assessed using the quadratic weighted Cohen’s kappa. Normality was evaluated with the Shapiro-Wilk test. Normally distributed continuous variables are presented as mean ± standard deviation, whereas non-normally distributed variables are reported as median (interquartile range). Homogeneity of variance was assessed using Levene’s test. Comparisons across the five models were conducted using one-way analysis of variance (ANOVA) for parametric variables and the Kruskal–Wallis H test for non-parametric variables. When an overall significant difference was detected, post hoc pairwise comparisons with Bonferroni correction were performed. A two-sided P value <0.05 was considered statistically significant.

## 3 Results

### 3.1 Output efficiency and structural characteristics of responses

A total of 150 model outputs were obtained in this study. Significant between-model differences were observed in Response times and key structural characteristics of the generated texts (Table 1; all P < 0.001). With respect to Response times, ChatGPT-5.2 had the shortest mean response times (17.94 ± 5.11), which were significantly faster than Gemini 3 Pro, DeepSeek-V3.1, Doubao, and Kimi K2 (all P < 0.001). Gemini 3 Pro (24.27 ± 3.75) was also significantly faster than DeepSeek-V3.1 (41.46 ± 3.22), Doubao (30.44 ± 5.64), and Kimi K2 (40.02 ± 4.67) (Tables 2–4; all P < 0.001). No significant difference in response times were found between DeepSeek-V3.1 and Kimi K2 (P = 0.223).

**TABLE 1:** Response times and textual output characteristics across five LLMs.

Models	Response times	Words	Characters	Paragraphs	Sentences	Tables
ChatGPT-5.2	17.94 ± 5.11	892.93 ± 206.10	1,053.53 ± 220.05	51.77 ± 5.16	64.60 ± 11.90	2.00 (1.00,2.00)
Gemini 3 pro	24.27 ± 3.75	1,064.47 ± 243.05	1,119.80 ± 212.97	38.03 ± 7.76	49.73 ± 9.37	1.00 (1.00,1.25)
DeepSeek-V3.1	41.46 ± 3.22	1,456.93 ± 224.99	1,672.67 ± 214.98	43.37 ± 9.80	77.60 ± 7.08	0.00 (0.00,0.00)
Doubao	30.44 ± 5.64	787.00 ± 269.01	830.63 ± 249.08	33.87 ± 4.11	41.20 ± 6.71	0.00 (0.00,1.00)
Kimi K2	40.02 ± 4.67	640.83 ± 252.95	790.87 ± 232.99	21.83 ± 7.58	32.73 ± 8.19	0.00 (0.00,1.00)
P-values	**<0.001**	**<0.001**	**<0.001**	**<0.001**	**<0.001**	**<0.001**

Data are presented as mean ± SD for normally distributed variables and as median (IQR) for non-normally distributed variables. Normality was assessed using the Shapiro–Wilk test, and homogeneity of variance was assessed using Levene’s test. Comparisons across groups were performed using one-way ANOVA for parametric variables and the Kruskal–Wallis H test for non-parametric variables. When an overall significant difference was detected, post hoc pairwise comparisons with Bonferroni correction were performed. Values in bold indicate statistical significance (*P* < 0.05).

**TABLE 2:** Comparative analysis of textual length: Words and Characters.

Characters\words	ChatGPT-5.2	Gemini 3 pro	DeepSeek-V3.1	Doubao	Kimi K2
ChatGPT-5.2	—	**0.040**	**<0.001**	0.430	**<0.001**
Gemini 3 pro	0.259	—	**<0.001**	**<0.001**	**<0.001**
DeepSeek-V3.1	**<0.001**	**<0.001**	—	**<0.001**	**<0.001**
Doubao	**0.002**	**<0.001**	**<0.001**	—	0.130
Kimi K2	**<0.001**	**<0.001**	**<0.001**	0.497	—

Pairwise comparisons were performed with Bonferroni correction after a significant overall test. The upper triangle shows adjusted P values for Words, and the lower triangle shows adjusted P values for Characters. Values in bold indicate statistical significance (*P* < 0.05).

**TABLE 3:** Comparative analysis of text segmentation: Paragraphs and Sentences.

Sentences\paragraphs	ChatGPT-5.2	Gemini 3 pro	DeepSeek-V3.1	Doubao	Kimi K2
ChatGPT-5.2	—	<0.001	<0.001	0.001	<0.001
Gemini 3 pro	**<0.001**	—	**0.040**	0.088	**<0.001**
DeepSeek-V3.1	**<0.001**	**<0.001**	—	**<0.001**	**<0.001**
Doubao	**<0.001**	**0.001**	**<0.001**	—	**<0.001**
Kimi K2	**<0.001**	**<0.001**	**<0.001**	**<0.001**	—

Pairwise comparisons were performed with Bonferroni correction after a significant overall test. The upper triangle shows adjusted P values for Paragraphs, and the lower triangle shows adjusted P values for Sentences. Values in bold indicate statistical significance (*P* < 0.05).

**TABLE 4:** Comparative analysis of speed and formatting: Response times and Tables.

Tables\response times	ChatGPT-5.2	Gemini 3 pro	DeepSeek-V3.1	Doubao	Kimi K2
ChatGPT-5.2	—	<0.001	<0.001	<0.001	<0.001
Gemini 3 pro	0.087	—	**<0.001**	**<0.001**	**<0.001**
DeepSeek-V3.1	**<0.001**	**<0.001**	—	**<0.001**	0.223
Doubao	**<0.001**	**0.001**	1.000	—	**<0.001**
Kimi K2	**<0.001**	**<0.001**	1.000	1.000	—

Pairwise comparisons were performed with Bonferroni correction after a significant overall test. The upper triangle shows adjusted P values for Response times, and the lower triangle shows adjusted P values for Tables. Values in bold indicate statistical significance (*P* < 0.05).

In terms of response length and structure, DeepSeek-V3.1 generated the largest outputs, with the highest Words (1,456.93 ± 224.99), Characters (1,672.67 ± 214.98), and Sentences (77.60 ± 7.08), which were significantly greater than those of the other models overall (Tables 2–4; most pairwise comparisons P < 0.001). In contrast, Kimi K2 produced the smallest outputs (Words 640.83 ± 252.95; Characters 790.87 ± 232.99; Sentences 32.73 ± 8.19) (Table 1). Notably, ChatGPT-5.2 and Doubao did not differ significantly in Words (P = 0.430), but they differed significantly in Characters, Paragraphs, and Sentences (P = 0.002, P = 0.001, and P < 0.001, respectively) (Tables 2–4). In addition, ChatGPT-5.2 yielded the highest number of Paragraphs (51.77 ± 5.16), which was significantly higher than that of most other models (Tables 2–4; most P < 0.001).

**FIGURE 2:**
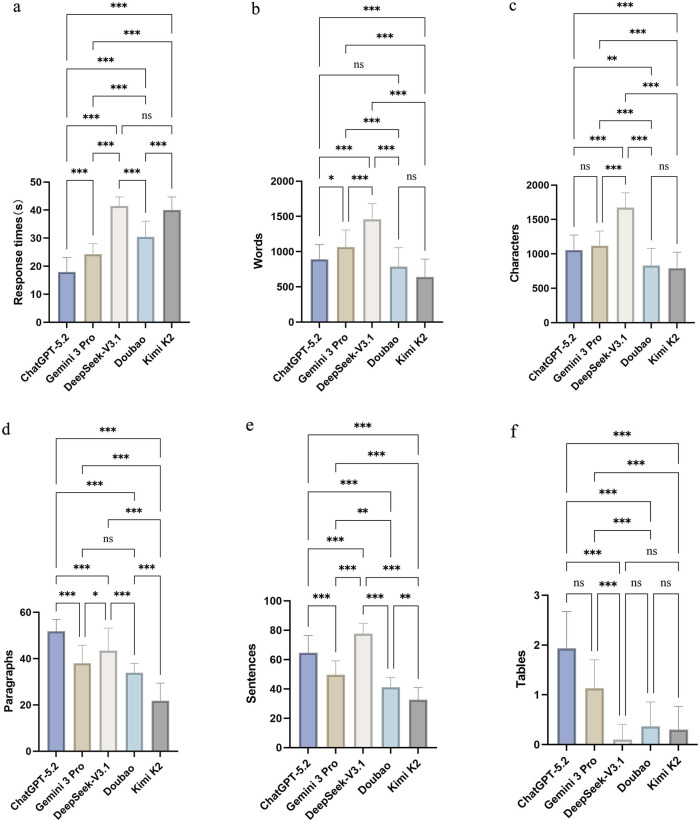
Response times and textual output characteristics across five large language models (LLMs). **(a)** Response times; **(b)** Words; **(c)** Characters; **(d)** Sentences; **(e)** Paragraphs; **(f)** Tables.

Regarding the propensity to generate Tables, ChatGPT-5.2 had the highest median number of tables [2.00 (1.00, 2.00)], followed by Gemini 3 Pro [1.00 (1.00, 1.25)], whereas DeepSeek-V3.1 produced no tables [0.00 (0.00, 0.00)]; Doubao and Kimi K2 also had a median of 0.00 tables (Table 1). Pairwise comparisons showed that ChatGPT-5.2 generated significantly more tables than DeepSeek-V3.1, Doubao, and Kimi K2 (all P < 0.001), whereas no significant differences were observed among DeepSeek-V3.1, Doubao, and Kimi K2 (all P = 1.000) (Tables 2–4). The difference in the number of tables between ChatGPT-5.2 and Gemini 3 Pro did not reach statistical significance (P = 0.087) (Figure 2).

### 3.2 Comparison of content quality scores across five domains

**FIGURE 3:**
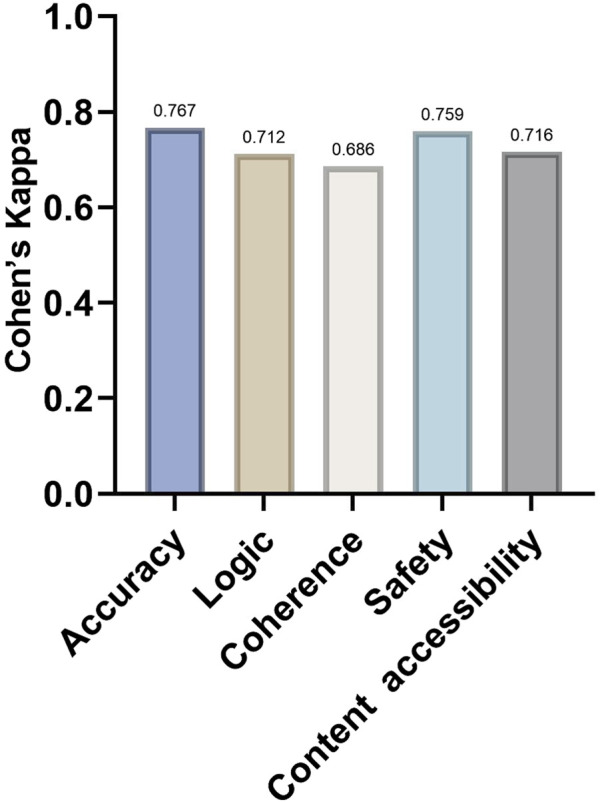
Quadratic weighted Cohen’s kappa across five evaluation dimensions.

Before comparing content quality across models, we first assessed inter-rater agreement between the two ophthalmologist reviewers, quadratic weighted Cohen’s kappa for Accuracy, Logic, Coherence, Safety, and Content accessibility were 0.767,0.712,0.686,0.759, and 0.716,respectively (Figure 3), indicating good inter-rater reliability and supporting subsequent between-model comparisons based on this rating framework. Significant between-model differences were observed in score distributions across all five domains (Accuracy: H = 41.15, P < 0.001; Logic: H = 32.95, P < 0.001; Coherence: H = 27.79, P < 0.001; Safety: H = 31.72, P < 0.001; Content accessibility: H = 41.33, P < 0.001) (Table 5).

**TABLE 5:** Comparison of five LLMs across five content quality dimensions.

Models	Accuracy	Logic	Coherence	Safety	Content accessibility
ChatGPT-5.2	4.00 (4.00, 5.00)	4.00 (4.00, 5.00)	4.00 (4.00, 5.00)	4.00 (4.00, 4.25)	4.50 (4.00, 5.00)
Gemini 3 pro	4.00 (4.00, 5.00)	4.00 (4.00, 5.00)	4.00 (4.00, 5.00)	4.00 (4.00, 5.00)	5.00 (4.00, 5.00)
DeepSeek-V3.1	4.00 (3.00, 4.00)	4.00 (3.00, 4.00)	4.00 (3.00, 4.25)	4.00 (3.00, 4.00)	4.00 (3.75, 4.00)
Doubao	4.00 (4.00, 4.00)	4.00 (3.00, 4.00)	4.00 (4.00, 4.25)	4.00 (3.75, 4.00)	4.00 (3.00, 4.00)
Kimi K2	3.50 (3.00, 4.00)	4.00 (2.75, 4.00)	3.50 (2.75, 4.00)	4.00 (3.00, 4.00)	4.00 (3.00, 4.00)
*H*-values	41.15	32.95	27.79	31.72	41.33
*P*-values	**<0.001**	**<0.001**	**<0.001**	**<0.001**	**<0.001**

Data are presented as median (IQR). Overall comparisons were performed using the Kruskal–Wallis H test. H-values and corresponding two-sided P values are shown. When an overall significant difference was detected, post hoc pairwise comparisons with Bonferroni correction were performed. Values in bold indicate statistical significance (*P* < 0.05).

Overall, ChatGPT-5.2 and Gemini 3 Pro achieved consistently high median scores across all domains. The median (interquartile range) scores were 4.00 (4.00, 5.00) for Accuracy, 4.00 (4.00, 5.00) for Logic, and 4.00 (4.00, 5.00) for Coherence in both models. For Safety, the median score was 4.00 (4.00, 4.25) for ChatGPT-5.2 and 4.00 (4.00, 5.00) for Gemini 3 Pro. For Content accessibility, Gemini 3 Pro achieved the highest median score of 5.00 (4.00, 5.00), whereas ChatGPT-5.2 scored 4.50 (4.00, 5.00) (Table 5). Pairwise comparisons indicated no statistically significant differences between ChatGPT-5.2 and Gemini 3 Pro across any domain (all P = 1.000) (Tables 6–10).

**TABLE 6:** Pairwise comparisons of Accuracy across five LLMs.

*Z*-values\*P*-values	ChatGPT-5.2	Gemini 3 pro	DeepSeek-V3.1	Doubao	Kimi K2
ChatGPT-5.2	—	1.000	**0.006**	0.203	**0.000**
Gemini 3 pro	0.000	—	**0.006**	0.203	**0.000**
DeepSeek-V3.1	3.421	3.421	—	1.000	0.652
Doubao	2.320	2.320	1.101	—	**0.032**
Kimi K2	5.265	5.265	1.844	2.945	—

Pairwise comparisons for Accuracy were performed with Bonferroni correction after a significant overall Kruskal–Wallis H test. The upper triangle presents adjusted P values, and the lower triangle presents the standardized test statistics (Z-values). Values in bold indicate statistical significance (*P* < 0.05).

**TABLE 7:** Pairwise comparisons of Logic across five LLMs.

*Z*-values\*P*-values	ChatGPT-5.2	Gemini 3 pro	DeepSeek-V3.1	Doubao	Kimi K2
ChatGPT-5.2	—	1.000	**0.003**	**0.003**	**0.000**
Gemini 3 pro	0.360	—	**0.012**	**0.011**	**0.001**
DeepSeek-V3.1	3.590	3.230	—	1.000	1.000
Doubao	3.613	3.253	0.023	—	1.000
Kimi K2	4.315	3.955	0.726	0.703 0.703	—

Pairwise comparisons for Logic were performed with Bonferroni correction after a significant overall Kruskal–Wallis H test. The upper triangle presents adjusted P values, and the lower triangle presents the standardized test statistics (*Z*-values). Values in bold indicate statistical significance (*P* < 0.05).

**TABLE 8:** Pairwise comparisons of Coherence across five LLMs.

*Z*-values\*P*-values	ChatGPT-5.2	Gemini 3 pro	DeepSeek-V3.1	Doubao	Kimi K2
ChatGPT-5.2	—	1.000	0.746	1.000	**0.001**
Gemini 3 pro	−0.825	—	0.091	0.396	**0.000**
DeepSeek-V3.1	1.783	2.608	—	1.000	0.244
Doubao	1.233	2.058	0.549	—	0.051
Kimi K2	4.033	4.858	2.250	2.800	—

Pairwise comparisons for Coherence were performed with Bonferroni correction after a significant overall Kruskal–Wallis H test. The upper triangle presents adjusted P values, and the lower triangle presents the standardized test statistics (*Z*-values). Values in bold indicate statistical significance (*P* < 0.05).

**TABLE 9:** Pairwise comparisons of Safety across five LLMs.

*Z*-values\*P*-values	ChatGPT-5.2	Gemini 3 pro	DeepSeek-V3.1	Doubao	Kimi K2
ChatGPT-5.2	—	1.000	**0.008**	0.143	**0.002**
Gemini 3 pro	−0.661	—	**0.001**	**0.019**	**0.000**
DeepSeek-V3.1	3.350	4.011	—	1.000	1.000
Doubao	2.450	3.111	0.900	—	1.000
Kimi K2	3.733	4.395	0.384	1.283	—

Pairwise comparisons for Safety were performed with Bonferroni correction after a significant overall Kruskal–Wallis H test. The upper triangle presents adjusted P values, and the lower triangle presents the standardized test statistics (*Z*-values). All P values were two-sided. Values in bold indicate statistical significance (*P* < 0.05).

**TABLE 10:** Pairwise comparisons of Content accessibility across five LLMs.

*Z*-values \ *P*-values	ChatGPT-5.2	Gemini 3 pro	DeepSeek-V3.1	Doubao	Kimi K2
ChatGPT-5.2	—	1.000	**0.010**	**0.006**	**0.000**
Gemini 3 pro	0.195	—	**0.005**	**0.003**	**0.000**
DeepSeek-V3.1	3.292	3.487	—	1.000	1.000
Doubao	3.425	3.620	0.133	—	1.000
Kimi K2	4.927	5.122	1.635	1.502	—

Pairwise comparisons for Content accessibility were performed with Bonferroni correction after a significant overall Kruskal–Wallis H test. The upper triangle presents adjusted P values, and the lower triangle presents the standardized test statistics (*Z*-values). Values in bold indicate statistical significance (*P* < 0.05).

In contrast, Kimi K2 showed overall lower score distributions in multiple domains (e.g., Accuracy 3.50 (3.00, 4.00) and Coherence 3.50 (2.75, 4.00)) (Table 5). Pairwise analyses demonstrated that ChatGPT-5.2 outperformed Kimi K2 across Accuracy, Logic, Coherence, Safety, and Content accessibility (all P < 0.001). Gemini 3 Pro also scored significantly higher than Kimi K2 across all five domains (all P < 0.001) (Tables 6–10).

**FIGURE 4:**
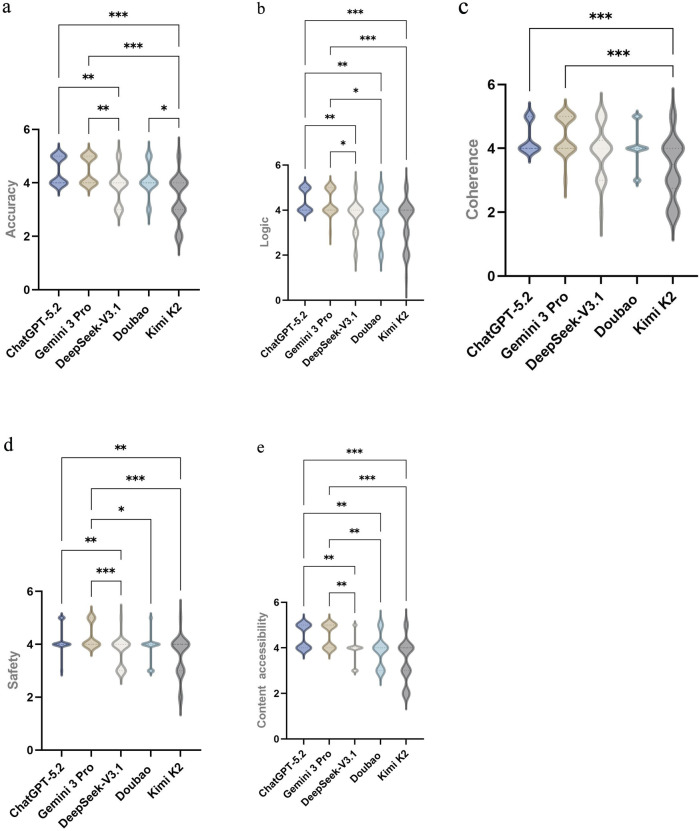
Pairwise comparisons of content-quality scores across five large language models (LLMs). **(a)** Accuracy comparison; **(b)** Logic; **(c)** Coherence; **(d)** Safety; **(e)** Content accessibility.

Among the domestic models, DeepSeek-V3.1 and Doubao did not differ significantly across any of the five domains (all P = 1.000) (Tables 6–10), suggesting comparable overall performance within the present evaluation framework. Relative to DeepSeek-V3.1, ChatGPT-5.2 achieved higher scores in Accuracy, Logic, Safety, and Content accessibility (P = 0.006, 0.003, 0.008, and 0.010, respectively), whereas no significant difference was observed for Coherence (P = 0.746). Comparisons between Gemini 3 Pro and DeepSeek-V3.1 showed a similar pattern, with higher scores for Gemini 3 Pro in Accuracy, Logic, Safety, and Content accessibility (P = 0.006, 0.012, 0.001, and 0.005, respectively), but not in Coherence (P = 0.091). In addition, ChatGPT-5.2 scored higher than Doubao in Logic and Content accessibility (P = 0.003,P = 0.006), while differences in Accuracy, Coherence, and Safety were not statistically significant. Gemini 3 Pro scored higher than Doubao in Logic, Safety, and Content accessibility (P = 0.011, 0.019, and 0.003, respectively) (Tables 6–10; Figure 4).

### 3.3 Correlations between output characteristics and content quality metrics

**FIGURE 5:**
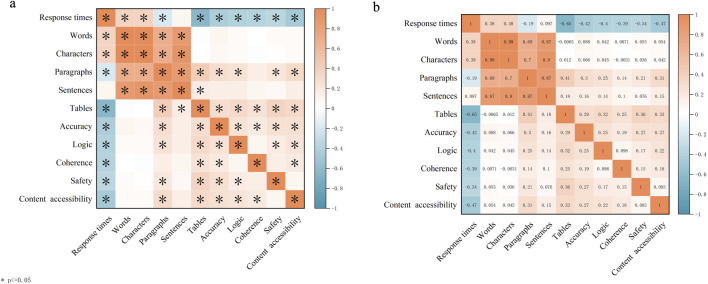
Correlations between response characteristics and content quality ratings.Correlation matrices were constructed using all model responses to examine associations between output features (Response times, Words, Characters, Paragraphs, Sentences, and Tables) and expert-rated quality domains (Accuracy, Logic, Coherence, Safety, and Content accessibility). Colors represent correlation coefficients (r), ranging from −1 (blue) to +1 (orange). **(a)** Heatmap with asterisks indicating statistically significant correlations (* *P*<0.05, two-sided). **(b)** Heatmap with corresponding r values annotated in each cell.

The correlation matrix between output characteristics and content quality metrics is presented in Figure 5. Overall, metrics reflecting response length and structure showed strong positive intercorrelations. The strongest association was observed between Words and Characters (r = 0.99). Words were also strongly correlated with Sentences (r = 0.87), as were Characters with Sentences (r = 0.90) and Paragraphs with Sentences (r = 0.87), indicating that different measures of response length and structural granularity were statistically highly consistent.

For efficiency-related metrics, Response times were negatively correlated with the number of Tables (r = −0.65) and were inversely associated with all five content quality domains (Accuracy r = −0.42; Logic r = −0.40; Coherence r = −0.39; Safety r = −0.34; Content accessibility r = −0.47). These findings suggest that longer response times did not translate into higher content quality ratings in the present study setting.

Notably, correlations between raw response length and quality ratings were generally weak. Words showed near-zero correlations with the five quality domains (r = 0.007–0.088), and similarly weak associations were observed for Characters (r = −0.003–0.066) and Sentences (r = 0.076–0.16). In contrast, more explicitly structured output features demonstrated more stable weak-to-moderate positive correlations with quality ratings. Specifically, Paragraphs were positively correlated with Accuracy (r = 0.30) and Content accessibility (r = 0.31). The number of Tables was also positively correlated with Safety (r = 0.36), Content accessibility (r = 0.33), and Logic (r = 0.32) (Figure 5).

Within the content quality domains, the five scores were generally weakly positively correlated with one another (e.g., Accuracy with Safety r = 0.27; Accuracy with Content accessibility r = 0.27; Logic with Content accessibility r = 0.22). This pattern suggests that although the domains are related, they are not fully overlapping, supporting the use of a multidimensional framework for comprehensive evaluation of model-generated responses (Figure 5).

## 4 Discussion

To our knowledge, this study provides a cross-sectional evaluation of consultation-style outputs generated by five mainstream Chinese conversational LLMs in the context of CC, a highly time-sensitive pediatric ophthalmology scenario in which caregiver decision-making is central. Overall, three main findings emerged. First, ChatGPT-5.2 and Gemini 3 Pro constituted the current practical reference benchmark in Accuracy and Safety, and they more frequently adopted structured information organization. Second, response length was not linearly associated with content quality: generating longer outputs did not yield higher accuracy or better content accessibility and may even increase caregivers’ cognitive burden. Third, structured outputs (i.e., clearer segmentation into Paragraphs and the use of Tables) showed more stable positive associations with Safety and Content accessibility, whereas Response times were generally negatively correlated with quality domains. Collectively, these results suggest that “slower” or “longer” responses do not necessarily indicate “better” or “safer” answers.

Prior research on medical LLMs has shown that LLMs can approach clinician-level performance on multidisciplinary medical benchmarks in terms of knowledge expression and reasoning (Singhal et al., 2023; Khatri and Gupta, 2025; Singhal et al., 2025). However, fluent responses may still be incomplete, overconfident, or potentially harmful (Griot et al., 2025), underscoring the need for structured evaluation, human review, and risk-stratified assessment before real-world deployment (Chen et al., 2024a; Gaber et al., 2025; Huo et al., 2025; Haltaufderheid et al., 2024). In ophthalmology, studies have reported that general-purpose models such as ChatGPT can achieve overall performance comparable to ophthalmologists for certain question types (Bernstein et al., 2023; Yılmaz and Doğan, 2024; Mihalache et al., 2023), yet their stability and safety-netting statements remain sensitive to the clinical context and question framing (Betzler et al., 2023; Chen et al., 2024b). Moreover, ophthalmology-specific models and evaluation studies have suggested that, for complex or time-sensitive problems, LLMs are prone to omissions at critical decision points—particularly regarding when urgent care is required and how to balance surgical timing against complication risks—thereby introducing downstream management risk (Wilhelm et al., 2023; Gupta et al., 2024; Cappellani et al., 2024). Our findings are consistent with this growing body of evidence: even when high-performing models demonstrate advantages in fluency and breadth of coverage, their outputs must incorporate explicit risk boundaries and triage-oriented guidance to align with the clinical realities of pediatric cataract care (Dihan et al., 2024).

CC involves not only a “sooner is better” window for visual development (Lenhart and Lambert, 2022), but also a complex long-term care continuum that includes scheduled follow-up, refractive correction, adherence to amblyopia therapy, and ongoing surveillance for complications (Repka et al., 2022; Yen et al., 2023). As a result, caregivers often experience pronounced information uncertainty and substantial caregiving burden after diagnosis (Chen et al., 2019). Qualitative evidence indicates that families urgently need guidance that is trustworthy, easy to understand, and readily revisitable during the interval between initial diagnosis and specialist evaluation (Du et al., 2025; De Lima et al., 2020). Data from the Infant Aphakia Treatment Study and related work further suggest that caregiving stress is particularly prominent during the early treatment period (Drews-Botsch et al., 2019). In addition, structured health education interventions for caregivers of children with CC have been shown to significantly alleviate caregiver anxiety and depressive symptoms and to improve subjective stress experiences, underscoring the practical value of information that is accessible, comprehensible, and actionable (Drews-Botsch et al., 2024). Therefore, prioritizing Content accessibility and Safety as core evaluation domains is well justified in this pediatric context.

Our correlation analyses provide further insight into the observed model differences. Words, Characters, and Sentences were highly intercorrelated, indicating that these measures primarily capture response “volume.” However, these volume-related metrics showed near-zero correlations with quality domains, suggesting that length alone, or the intuitive impression that “more information” implies higher reliability, is an unreliable proxy for response quality, consistent with prior reports (Vladic et al., 2025; Bulla et al., 2025). In contrast, more structured output features, such as Paragraphs and Tables, showed more stable positive associations with Safety and Content accessibility. This pattern may reflect the fact that structured formats better support information organization aligned with caregiver decision-making needs, including stepwise action recommendations, checklist-style risk warnings, and concise postoperative home-care instructions (Shoemaker et al., 2014; Sayfi et al., 2023; Ayers et al., 2023). Meanwhile, Response times were negatively correlated with multiple quality domains, indicating that, under our study setting, slower generation did not translate into safer or more accurate responses and suggesting that response latency should not be treated as a surrogate marker of quality in future evaluations (Friederichs et al., 2023).

Readability challenges in pediatric ophthalmology education materials have been repeatedly documented, with online ophthalmic patient education resources frequently exceeding recommended reading levels and thereby increasing the risk of misunderstanding (Huang et al., 2015). LLMs have been proposed as tools to help generate more readable, public-facing health information, but concerns remain regarding inconsistency and the potential for misleading content, highlighting the need for human oversight and structured guardrails (Tam et al., 2024). Recent work focusing on pediatric cataract patient education materials further suggests that LLM-based rewriting can improve readability and understandability, yet clinical expert review remains necessary to verify factual accuracy and delineate appropriate risk boundaries (Qiu et al., 2025). These observations align with our findings: although higher-performing models appear to offer advantages in Content accessibility, their outputs should still be constrained by standardized, safety-oriented templates. In practice, this could involve first stating clear “when to seek urgent care” thresholds, then outlining what families can do at home, and finally providing follow-up plans and warning symptoms, thereby reducing the likelihood of misuse.

Building on our findings and prior evidence, we recommend that future applications of LLMs in pediatric cataract health education and consultation support adopt risk-centered output standards. For any questions involving surgical timing, abnormal signs, or follow-up intervals, LLMs should be required to explicitly provide red-flag symptoms and clear thresholds for seeking medical care (Qiu et al., 2025; Edwards et al., 2022), while avoiding vague “watchful waiting” recommendations. Structured presentation formats, including bullet points, checklists, and table-based summaries, should be encouraged to enhance Content accessibility and actionability. Continuous monitoring should be implemented using multidimensional human evaluation frameworks such as QUEST, and platform-level quality review and iterative updating mechanisms should be established (Angus et al., 2025). In this setting, LLMs should be positioned as supportive tools for health education and care-seeking guidance rather than substitutes for clinical diagnosis. In parallel, governance strategies should address privacy protection, accountability and liability, and potential biases (Ayers et al., 2023; Duffourc and Gerke, 2023; Minssen et al., 2023).

Several limitations should be noted. First, this was a cross-sectional evaluation conducted within a single time window, and model performance may vary with version updates, platform policies, and runtime environments. Repeated assessments across multiple time points are needed to confirm the stability and reproducibility of our findings. Second, we adopted a zero-shot, single-turn question–answering setting without case-specific details or multi-turn follow-up, which may under- or overestimate performance under real-world interactive use. Future work should incorporate standardized case vignettes and expand the task set to multi-turn dialogue scenarios to better simulate actual consultations. Third, although inter-rater reliability was good, the expert ratings remain subjective, and the question set was developed based on expert experience, limiting external representativeness. Importantly, the “content accessibility” domain in this study reflects expert-rated comprehensibility/readability rather than direct end-user usability or comprehension testing among parents/caregivers (the intended users). Therefore, responses judged as “accessible” or “safe” by ophthalmologists may still be perceived as unclear, insufficient, or not adequately anxiety-relieving by families without medical backgrounds. Future studies should incorporate guideline-based objective concordance checks and fact verification, and include patient- and caregiver-centered evaluations (e.g., comprehension testing, usability surveys, perceived usefulness, anxiety alleviation, and actionability assessments) to improve verifiability and external validity.

## 5 Conclusion

This study demonstrates substantial performance differences among five mainstream LLMs in CC consultation scenarios. Gemini 3 Pro and ChatGPT-5.2 showed more stable performance in Accuracy and Safety and may serve as current reference benchmarks. Notably, longer responses did not necessarily indicate higher quality; instead, structured presentation was more consistently associated with better comprehensibility and more appropriate safety-netting. Beyond general counseling, our findings highlight a key translational challenge at the interface of AI and cellular/developmental ophthalmology: whether LLMs can reliably translate lens developmental pathology and etiologic/genetic concepts into safe, actionable parent education. LLMs have potential to support pediatric cataract health education and clinician-delivered counseling, but reliability and generalizability should be strengthened through repeated multi-time-point assessments, multi-turn dialogue evaluations, and incorporation of objective verification metrics (e.g., checklist-based auditing of pathology/genetics coverage and red-flag guidance) to enable safer deployment and governance.

## Data Availability

The original contributions presented in the study are included in the article/supplementary material, further inquiries can be directed to the corresponding authors.
